# Endothelial Senescence and the Chronic Vascular Diseases: Challenges and Therapeutic Opportunities in Atherosclerosis

**DOI:** 10.3390/jpm12020215

**Published:** 2022-02-04

**Authors:** Rafael Ramírez, Noemi Ceprian, Andrea Figuer, Gemma Valera, Guillermo Bodega, Matilde Alique, Julia Carracedo

**Affiliations:** 1 Departamento de Biología de Sistemas, Universidad de Alcalá, 28871 Alcalá de Henares, Madrid, Spain/Instituto Ramón y Cajal de Investigación Sanitaria (IRYCIS), 28034 Madrid, Spain; manuel.ramirez@uah.es (R.R.); andrea.figuer@salud.madrid.org (A.F.); 2 Departamento de Genética, Fisiología y Microbiología, Facultad de Ciencias Biológicas, Universidad Complutense de Madrid/Instituto de Investigación Sanitaria Hospital 12 de Octubre (imas12), 28040 Madrid, Spain; nceprian@ucm.es (N.C.); gemmavaar@hotmail.com (G.V.); 3 Departamento de Biomedicina y Biotecnología, Universidad de Alcalá, 28871 Alcalá de Henares, Madrid, Spain; guillermo.bodega@uah.es

**Keywords:** atherosclerosis, cellular senescence, endothelial senescence, extracellular vesicles, inflammation, senolityc

## Abstract

Atherosclerosis is probably one of the paradigms of disease linked to aging. Underlying the physiopathology of atherosclerosis are cellular senescence, oxidative stress, and inflammation. These factors are increased in the elderly and from chronic disease patients. Elevated levels of oxidative stress affect cellular function and metabolism, inducing senescence. This senescence modifies the cell phenotype into a senescent secretory phenotype. This phenotype activates immune cells, leading to chronic systemic inflammation. Moreover, due to their secretory phenotype, senescence cells present an increased release of highlighted extracellular vesicles that will change nearby/neighborhood cells and paracrine signaling. For this reason, searching for specific senescent cell biomarkers and therapies against the development/killing of senescent cells has become relevant. Recently, senomorphic and senolityc drugs have become relevant in slowing down or eliminating senescence cells. However, even though they have shown promising results in experimental studies, their clinical use is still yet to be determined.

## 1. Introduction

Atherosclerosis is the most common cardiovascular system disorder characterized by atheroma’s plaque formation on arterial walls [1]. Even though atherosclerosis is commonly associated with large and medium arteries damage [2], it affects all vascular cells. Clinical symptoms appear when the affected vessel is coronary, carotid, or cerebral. Artery plaque formation is a progressive disorder initiated by damage accumulation in the artery and influenced by cardiovascular risk factors [1], including age, which is a universal factor for arteriosclerosis development [3].

The incidence of most common chronic diseases, such as atherosclerosis, neurodegenerative disease, cancer, or diabetes, increases in people over 65 years old [4,5]. In the following years, these chronic diseases, known as age-related diseases, are expected to increase due to lifespan increases [6,7]. One common hallmark of these age-related diseases is cellular senescence [8]. In this regard, several clinical and experimental studies have suggested that the physiopathology of this age-related disease is associated with the physiological process of cellular senescence, which is activated in damage associated with stressful situations in all types of cells, as shown in tumor formation [9]. The aging process is associated with a frailty situation in which old adults impair the physiological homeostatic system, which cannot respond successfully to stressful situations. This frailty state is reached when tissues and systems fail because their cells have become senescent due to damage accumulation over the years or by damaging agents in chronic pathologies [3].

This review aims to deepen the perspective of atherosclerosis as a disease associated with cellular senescence; not only as a compilation of studies, but the cellular senescence concept can change the paradigm of age as a nonmodifiable risk factor. Likewise, knowing how and why the senescent cell acts as a pathophysiological mechanism in developing age-specific diseases offers new approaches to early diagnosis and the possibility of new therapeutic strategies based on the control of cellular senescence.

## 2. Cellular Senescence

Atherosclerosis is a disease with a multifactorial origin. For many years, atherosclerosis was considered a consequence of “modifiable” risk factors like smoking, obesity, high blood pressure, hypercholesterolemia, and other elements associated with pathologies, among which diabetes or chronic kidney disease stand out [8]. The prevention and correction of these modifiable risk factors is the attributed cause of the improvement in the tendencies of the cardiovascular morbimortality rates observed in recent years [10]. Moreover, atherosclerosis is associated with no modifiable factors like age or sex. Nowadays, age is one of the main risk factors due to the drastic decrease in the prevalence of modifiable risk factors [10]. However, several studies [11,12,13,14] have proven that physiological aging is not a risk factor. Furthermore, the physiopathological mechanism of atherosclerosis is associated with the cellular senescence process, which is related to aging, but also, it can be accelerated by modifiable risk factors [11].

Cellular senescence is an essential defense program of the organism against irreparable damages that could endanger the transmission of genetic information and ensure adequate cellular activity [12]. Cellular senescence is characterized by morphological and metabolic changes, chromatin reorganization, and modified genetic expression. This status provokes an interruption of the replicative activity and resistance to apoptosis [15]. In addition, several cellular damage inductors can cause cellular senescence, and it is commonly associated with an increase in cellular oxidative stress. Even though these facts identify cellular senescence as a physiological mechanism to prevent tumor development in adults initially [15], its long-term effects are detrimental in tumor formation and development [16], and it is considered one of the hallmarks of cancer [17]. What is more, it plays a crucial role in embryogenesis and fetal development and participates in tissue regeneration and scarring [18].

One of the acquired characteristics of senescent cells is the acquisition of a proinflammatory phenotype called the senescence-associated secretory phenotype (SASP). This phenotype consists of a dysregulated production of proinflammatory cytokines, chemokines, growth factors, and proteases [19]. On the one hand, this phenotype could be considered an essential activator of an efficient immune response that controls the cellular senescence process. On the other hand, SASP could be one of the primary mechanisms that turn cellular senescence into a physiopathological mechanism in developing age-related diseases [20].

When the immune response is inefficient, they cannot satisfactorily eliminate senescence cells, therefore accumulating [21]. The SASP activity could be harmful, causing damage in the surrounding tissue and extending the senescence process to other cells and tissues in a process referred to as the senescence-induced bystander effect [20]. In addition, cellular senescence expanded the autocrine and paracrine responses in this process, affecting immunocompetent cells and other distant structures.

### 2.1. Factors That Induce Endothelial Senescence

**Figure 1:**
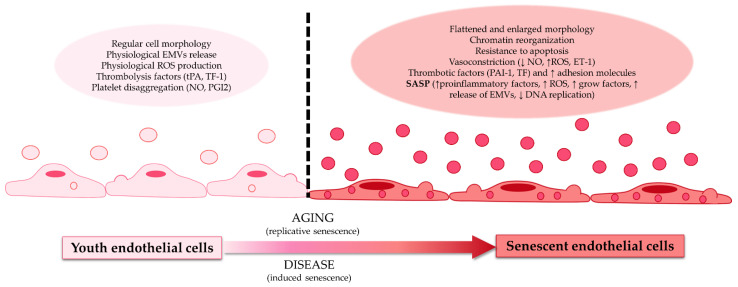
Differences between youth endothelial cells and senescent endothelial cells. Youth endothelial cells presented regular morphology, physiological release, and the production of extracellular microvesicles (EMVs), reactive oxygen species (ROS), a thrombolysis factor (such as tissue factor 1 (TF-1) or tissue plasminogen activator (tPA)), and platelet disaggregation factors (like nitric oxide (NO) or prostaglandin I 2 (PGI2)). These endothelial cells transform with age (replicative senescence) or with disease (induced senescence) into senescent endothelial cells. Senescent endothelial cells have a flattened and enlarged morphology, presenting chromatin reorganization; resistance to apoptosis; vasoconstriction factors (reduction of NO, increase of ROS, and endothelin receptor 1 (ET-1)); thrombotic factors (such as tissue factor (TF) and plasminogen activator inhibitor-1 (PAI-1)); an increase of adhesion molecules; and the senescence-associated secretory phenotype (increase secretion of proinflammatory factors, ROS, growth factors, EMVs, and diminution of DNA replication).

Numerous amounts of evidence hold that one of the first steps in atherosclerosis development is vascular endothelial damage [20]. The vascular endothelium is essential in maintaining systemic flow homeostasis and perfusion due to the ability of endothelial cells to regulate the vascular diameter and tone and maintain the blood flow of immunocompetent cells, platelets, and other elements [22]. In addition, the functional physiological activity loss of the endothelial cell is associated with cellular aging, and it is considered a pathogenic mechanism in the initial steps of endothelial damage and atherosclerosis. Due to that, cell damage and endothelial dysfunction are considered early indicators of atherosclerosis development [22] (Figure 1).

Cellular senescence is a heterogenic process specific to the different cell types and tissues. For example, senescent vascular endothelial cells and vascular dysfunction may play a critical mechanism in explaining the increase of cardiovascular disease with aging [23,24].

In vivo senescent endothelial cells of animal models and human tissues have been identified in different pathological conditions [25]. For example, senescence-associated beta-galactosidase (SA-β-gal) dye [26] is a commonly used marker to recognize cellular senescence, and it has revealed an increase of senescent endothelial cells in diabetic Zucker rats [27,28]. The same phenomenon was observed in animals of advanced age, with increased molecules associated with cellular senescence-like p53, p21, and p16 [28,29]. Senescent endothelial cells SA-β-gal-positive have also been found in the human coronary arteries, aortic atherosclerotic plaques [30,31], and adipocyte tissue of obese human subjects [32]. Furthermore, senescent endothelial cells show structural and functional changes, with proinflammatory, prothrombotic, and vasoconstrictive phenotypes [23].

Genotoxic, oxidative, and metabolic stresses lead to inadequate DNA reparation, resulting in DNA damage [33], essential in endothelial senescence. Additionally, cells’ inability in proliferation, cellular survival, apoptosis, or autophagy processes also contributes to inadequate DNA reparation [33]. In conclusion, the impossibility of repairing the DNA contributes to vascular aging and the development of cardiovascular diseases [34,35].

Another process that defines cellular senescence is the mitochondrial function alteration, which implies the liberation of oxidative species produced by mitochondria [36,37]. Oxidative stress causes oxidative damage in DNA, lipids, and proteins, and these damaged molecules can promote cellular senescence of the vascular endothelium [37]. In the vascular endothelium, the hydrogen peroxide levels are the main form of endogen oxygen reactive species. Its levels are increased mainly by the deterioration and the impaired function of the antioxidant systems [38]. This fact has been demonstrated in vitro in a culture of aged cells, in which the reactive oxygen species increase was associated with telomere shortening [39]. The telomere length is, in fact, one of the critical aspects implicated in the cellular senescence process [39], proven by the observation of telomeric shortening in senescent endothelial cells [40]. In the endothelial cells from human aortas, telomere shortening induces senescence, while telomerase induction restores the cell function [31]. Additionally, the telomere length is shorter in cells with atherosclerotic lesions [40].

The senescent cells actively produce a complex “secretome”, previously defined as SASP [14,41,42]. The SASP consists of chemokines; proinflammatory cytokines (IL-1, IL-6, TNF-α, TFG-β, etc.); and proteases. When senescent cells accumulate, the liberation of these elements generates a persistent low degree of inflammation. To avoid it, the immune cells (monocytes, macrophages, natural killer cells, and T-cytotoxic lymphocytes) eliminate senescent cells [43]. Either way, the inflammation can become a chronic phenomenon due to the perpetuation of the release of proinflammatory factors caused by the accumulation of senescent cells. The higher number of senescent cells is generated by increased production or a deficient elimination of these cells by the immune system, because the immune cells are also susceptible to senescence [21,44].

### 2.2. Low Degree Chronic Systemic Inflammation as a Key Risk Factor of Endothelial Senescence

The relevance of the classical cardiovascular risk factors (high blood pressure, diabetes mellitus, dyslipidemia, smoking, and a sedentary lifestyle) on endothelial damage development and atherosclerosis is well-known. Furthermore, these factors increase oxidative stress, which induces endothelial senescence [45]. For that reason, lifestyle and therapies that increase antioxidants are extraordinary measures to avoid endothelial senescence and atherosclerosis [46,47].

Besides oxidative stress, inflammation represents another common pathogenic mechanism in developing endothelial damage and atherosclerosis in physiological aging and chronic diseases. These last usually appear together and give feedback to each other. In addition, high levels of oxidative stress generate endothelial damage, which recruits immune cells, which will produce oxidative and inflammatory compounds, affecting the endothelium and increasing the damage in a vicious circle [48].

In general, inflammation is an auto time-limited physiological response against aggressions led by immunocompetent cells through the secretion of cytokines, chemokines, and growth factors [20]. Physiological inflammation is an acute response to a wound, aggression, or pathogen. It stops once its cause is resolved, which usually occurs without any consequence or, occasionally, with minimum changes associated with a scarring process. However, if the detrimental cause that originates the acute inflammatory response is not controlled and eliminated, the inflammatory response can become chronic, persistent, and prolonged in time. Both inflammations usually cause destruction and fibrotic reparation in the tissue areas where the aggression is localized. However, the commonly observed inflammation in elderly or chronic pathologies, like diabetes or chronic kidney disease, does not resemble the previously described acute and chronic inflammation. In those cases, inflammation is a systemic process that is not limited to a restricted area, and it usually manifests as a moderated increase of cytokines and other inflammatory compounds. It is rare to observe a substantial increase in the neutrophil and lymphocyte counts, and it appears that the most commonly affected cell type is the monocyte [49]. This chronic low-degree systemic inflammation (SI) results from a process denominated inflammaging. Several studies suggest that inflammaging is a crucial pathogenic mechanism in multiple age-related diseases, including endothelial damage and atherosclerosis [50].

SI is a generalized process with specific inflammatory mediators [49]. The characteristics of this SI, systemic and low-grade, cannot be identified with a specific injury or loss of functionality in a determined tissue, which is a feature of acute and chronic inflammation. On the contrary, the damage generated by SI provokes deterioration of the general state by poorly described mechanisms. Generalized deterioration favors tumor onset processes and degenerative diseases like cardiovascular diseases [50].

Recently, SI has been proposed as an adaptive response associated with the aging process [49]. Still, it can occur in pathological conditions like chronic kidney disease, inducing reiterative activation of the immunocompetent cells [50,51,52,53,54]. In this regard, recent studies suggest that this process could be related to dysfunctional hematopoiesis, named clonal hematopoiesis (CH) of indeterminate potential (CHIP). In CHIP, the inflammatory environment in the bone marrow led to the generation of malignant clones and inflammation [55,56,57].

## 3. Extracellular Vesicles and Endothelial Senescence

SAPS characteristic molecules have a critical role in the capacity of senescent cells to communicate (damage, repair, or elimination signal) local or at a distance, even though knowledge of the SAPS is still limited. Extracellular vesicles (EVs) may play an essential role in this signaling mechanism. EVs act as vehicles of molecules, protecting them from degradation by enzymes or other processes that neutralize their signaling capacity. EVs’ cargo is proteins, lipids, and nucleic acids, among which are microRNAs (miRNAs) [58,59,60]. SASP proteins transported in EVs could also be distinct for each senescent phenotype.

EVs are a local and systemic signaling system and have a relevant role in the function and homeostasis of the organism [60]. EVs are produced by most cells in the body, including endothelial cells. They are part of normal cell function, although they increase under cellular stress, apoptosis, or altered cell viability [22,61,62].

EVs have been closely linked to pathological processes such as inflammation, fibrosis, thrombosis, adhesion, immune suppression, growth, and regeneration [63]. In addition, they are involved in the development of vascular calcification as a consequence of an accelerated vascular aging state [43]. Lehmann et al. (2008) described an increase in EV secretion associated with senescence for the first time [64]. This increase constitutes a general feature of cellular senescence observed in fibroblasts, epithelial cells, cancer cells, and endothelial cells [64,65]. DNA-damaging factors significantly increase EV secretion in most cases, impairing the regenerative capacity of endothelial cells and, therefore, decreasing their cell migration capacity and their potential to form vascular structures [66]. In addition, increased endothelial cell-derived EVs are associated with endothelial dysfunction produced by uremia and persistent inflammation in patients with chronic kidney disease [67].

**Figure 2:**
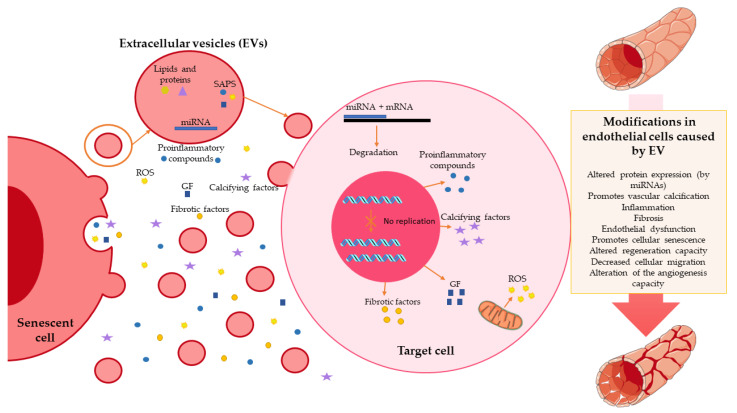
Role of extracellular vesicles in endothelial senescence. Senescence cells release extracellular vesicles that contain multiple factors, such as miRNA; lipids; proteins; and other substances related to senescence-associated secretory phenotype (SAPS), such as proinflammatory compounds, reactive oxygen species (ROS), or growth factors (GF). These compounds reach the target cell and exert multiple functions: alteration of the protein expression exerted by miRNAs; alteration of cell replication; increased production; and the release of proinflammatory, fibrotic, calcifying factors, GF, and ROS. These promote vascular calcification, inflammation, cellular senescence, endothelial dysfunction, and fibrosis.

Moreover, their content may also be altered, with the capacity to participate in endothelial dysfunction, fibrosis, and vascular calcification [68,69]. Additionally, there is a deregulation of the expression of specific miRNAs (such as miRNA-146b-5p and miRNA-223-3p) with cellular senescence. This change can also be contained in EVs and alter the expression of many proteins in the target cell [70] (Figure 2).

## 4. Target Endothelial Senescence: A Promising Therapeutic Opportunity

Aging was initially described as a nonmodifiable cardiovascular risk factor. It leads to multiple therapeutical strategies to prevent the mechanisms that unleash it [71]. When the increase of oxidative stress and inflammation was discovered as the leading causes of endothelial dysfunction, preventing them became the main priority in healthcare and society. Exercise, caloric restriction, and antioxidant ingestion have become healthy habits in our community to avoid cardiovascular diseases, such as atherosclerosis, preventing an increase in oxidative stress and endothelial damage associated with aging and disease. Although these and other therapies with similar targets have shown potential roles in preventing, or at least slowing down, vascular damage associated with cellular senescence, epidemics of diseases such as atherosclerosis in developed countries require additional effort. In our opinion, the priority should be the search for diagnostic biomarkers of patients at risk of developing aging-related diseases, as well as specific therapeutical options for preventing or eliminating senescence cells in elder subjects or patients with chronic disease.

Even though senescent cells have common molecular, biochemical, and functional characteristics, such as a proinflammatory activity associated with the SAPS phenotype [42], the regulation of epigenetic mechanisms of danger, or alterations in proteostasis [42,72,73,74], there is not currently a universal biomarker with a truly clinical appliance for identifying senescence cells.

Concerning the use of senescent cells as a therapeutic target, in recent years, therapies have been promoted to slow down SAPS activity with senomorphic drugs or eliminate senescent cells with so-called senolytic drugs [74,75,76]. Indeed, these therapies are based on the fact that senescent cells show an exacerbated SAPS activity, which, in many aging-associated diseases, seems to modulate in an autocrine way the activity of the senescent cell but, also, to extend the process of cellular senescence and, with it, the damage in a paracrine and endocrine way through epigenetic mechanisms [77]. Thus, drugs such as rapamycin, ruxolitinib, glucocorticoids, or metformin have been developed to inhibit senescence intracellular mediators (kinases, mTOR, etc.) or limit the secretory activity associated with the SAPS phenotype [44,78]. However, these therapies that have been effective in experimental models present limitations for their translation to clinical use, mainly associated with the side effects beyond their senomorphic properties. The second major antisenescence therapeutic wave based on senolytic drugs was probably in 2015, when dasatinib plus quercetin was used to promote the selective apoptosis of senescent cells in old mice [79].

In the 1990s, Wang et al. demonstrated that one of the characteristics of senescent cells such as fibroblasts was their resistance to apoptosis linked to the genetic and metabolic modifications that these cells undergo [80]. This peculiarity was used to generate drug combinations that promoted the selective apoptosis of senescent cells to limit the damage caused by them. Occasionally, these eliminated cells could be replaced by new cells differentiated from healthy progenitor cells [81].

From this description of senolytic drugs, an avalanche of new therapeutic combinations, small molecules, peptides, antibodies, or cell therapies with chimeric receptors such as chimeric antigen receptor T-cell (CAR-T) therapies appear as potential senolytic therapies in different pathologies. In addition, there have been numerous clinical trials initiated based on senolytics to treat age-related diseases [5,75].

**Figure 3:**
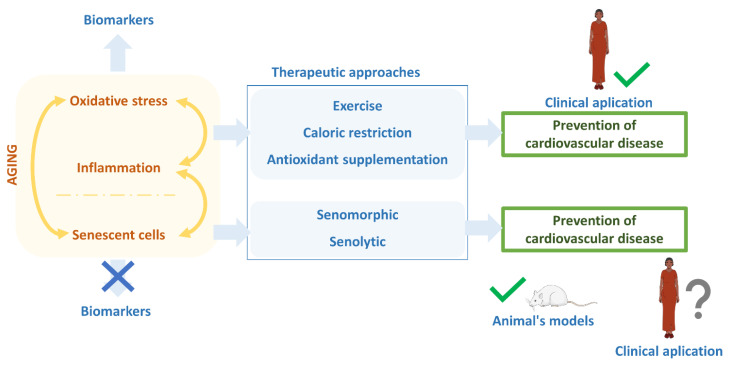
Estate of antiaging therapies. The aging process is characterized by increased oxidative stress, inflammation, and senescent cells, giving feedback to each other. Lowing oxidative stress and inflammation, which have biomarkers to know their state, are currently the best therapeutical options for reducing the aging rate and preventing age-related diseases like cardiovascular disease. For that, exercise, caloric restriction, or antioxidant supplementation have been the ideal therapeutic actions, even though the incidence of cardiovascular disease is still high in developed countries. Recently, the focus has been on reducing or eliminating senescent cells using senomorphic and senolytics drugs. Despite the lack of precise biomarkers, the results of these senolytics and senomorphics are auspicious in experimental animals. However, their actual clinical application still needs to be tested.

Concerning atherosclerosis, experimental studies have also emerged proposing the use of senolytic drugs as a potential therapy. For example, senolytics prevent or at least slow vascular dysfunction and atherosclerosis [82,83]. However, in a recent study, the results obtained in in vitro models of vascular smooth muscle cells limited these expectations [84] (Figure 3).

In this sense, all these studies conclude that a promising career has begun based on cellular senescence as a pathogenic mechanism in atherosclerosis and in many other aging-associated diseases, which has changed the paradigm that we currently face. In addition to preventing the development of aging-related illness through the risk factors associated, it is necessary to identify senescent biomarkers that allow us to identify patients at risk of developing pathologies such as atherosclerosis, where the damage is linked to senescent cell accumulation. Nevertheless, these biomarkers must be specific for the cells involved in the damaging process from clinical therapeutical use. However, some molecules such as determined genes, p16 proteins, and the beta-galactosidase marker may not be helpful from a clinical perspective, because they are part of the physiological machinery activity in most of the organism’s cells.
